# Combined Multi-Atlas and Multi-Layer Perception for Alzheimer's Disease Classification

**DOI:** 10.3389/fnagi.2022.891433

**Published:** 2022-06-01

**Authors:** Xin Hong, Kaifeng Huang, Jie Lin, Xiaoyan Ye, Guoxiang Wu, Longfei Chen, E. Chen, Siyu Zhao

**Affiliations:** ^1^College of Computer Science and Technology, Huaqiao University, Xiamen, China; ^2^Key Laboratory of Computer Vision and Machine Learning (Huaqiao University), Fujian Province University, Xiamen, China; ^3^Fuzhou Comvee Network and Technology Co., Ltd, Fuzhou, China; ^4^College of Foreign Languages, Huaqiao University, Quanzhou, China; ^5^Department of Neurology, The First Affiliated Hospital of Fujian Medical University, Fuzhou, China; ^6^Department of Neurosurgery, Zhongshan Hospital Affiliated to Xiamen University, Xiamen, China

**Keywords:** atlas, multi-layer perceptron, Alzheimer's disease, classification, PRCV competition

## Abstract

Alzheimer's disease (AD) is a progressive and irreversible neurodegenerative disease. To distinguish the stage of the disease, AD classification technology challenge has been proposed in Pattern Recognition and Computer Vision 2021 (PRCV 2021) which provides the gray volume and average cortical thickness data extracted in multiple atlases from magnetic resonance imaging (MRI). Traditional methods either train with convolutional neural network (CNN) by MRI data to adapt the spatial features of images or train with recurrent neural network (RNN) by temporal features to predict the next stage. However, the morphological features from the challenge have been extracted into discrete values. We present a multi-atlases multi-layer perceptron (MAMLP) approach to deal with the relationship between morphological features and the stage of the disease. The model consists of multiple multi-layer perceptron (MLP) modules, and morphological features extracted from different atlases will be classified by different MLP modules. The final vote of all classification results obtains the predicted disease stage. Firstly, to preserve the diversity of brain features, the most representative atlases are chosen from groups of similar atlases, and one atlas is selected in each group. Secondly, each atlas is fed into one MLP to fetch the score of the classification. Thirdly, to obtain more stable results, scores from different atlases are combined to vote the result of the classification. Based on this approach, we rank 10th among 373 teams in the challenge. The results of the experiment indicate as follows: (1) Group selection of atlas reduces the number of features required without reducing the accuracy of the model; (2) The MLP architecture achieves better performance than CNN and RNN networks in morphological features; and (3) Compared with other networks, the combination of multiple MLP networks has faster convergence of about 40% and makes the classification more stable.

## Introduction

Alzheimer's disease (AD) is a common neural degenerative disease, from which 60 to 70% of senile patients with dementia suffer (Jagust, 2013). A feature of AD is the damage induced by the irreversible and progressive cognitive function of human brains. It is continuously progressing when a normal-control (NC) gradually becomes a patient with AD. Mild cognitive impairment (MCI) is the early disease-developing stage (Reiman et al., 2010). Therefore, being able to correctly represent the disease-developing stage a patient is in helps in diagnosing and slowing the process of the disease. Over time, the condition of AD is often accompanied by brain atrophy. Recently, in Pattern Recognition and Computer Vision 2021 (PRCV 2021), the AD classification technology challenge^1^ provided a dataset from multiple atlas partitions and extracted volume features. This dataset is used for three classification tasks of NC/MCI/AD. The data of each sample in the dataset consists of brain gray matter volume and average cortical thickness that are extracted from multiple atlases.

The AD classification frameworks directly analyze the patterns in neuroimaging data of AD/MCI/NC subjects. In addition, the classification framework is comprised of multi-components: feature extraction, feature selection, dimensionality downsampling, and feature-based classification. According to the PRCV 2021, the task of the challenge is to do the three classifications of patients. Over the past decade, the cortical thickness, voxel-wise, and hippocampal morphological features of sMRI were used to diagnose AD (Jagust, 2013). After jointly aligning whole-brain image data to associate each brain voxel, voxel features have extracted a vector with multiple scalar measurements. Gray matter voxels are used for input features and trained in the support vector machine (SVM) classifier to classify AD and NC categories (Klöppel et al., 2008). To improve the performance of the model, the researchers used a 3D CNN to make predictions about the stage of the disease that the AD patient was in based on MRI (Bron et al., 2015). In some work, researchers have also improved the accuracy of classification by pre-training or providing model complexity (Payan and Montana, 2015; Korolev et al., 2017). In the competition, most of the better-performing teams have optimized their methods based on the multi-layer perceptron (MLP) architecture. The adjustments on the network are, broadly, as follows: combining MLP with attention mechanisms, adjusting the depth of the MLP network, combining multiple networks for data processing, etc. For the processing of datasets, some teams filtered data based on the characteristics of the atlas or supplemented the data with interpolation.

Since comparative evaluations of these feature extraction techniques reveal several limitations for classifying AD, we present a multi-atlas multi-layer perceptron (MAMLP) approach to a one-dimensional long vector data extracted from multiple atlases. Compared to the CNN and rerrent neural network (RNN) methods, our method converges faster and has higher accuracy during the training process. A network composed of multiple MLP modules achieves higher accuracy in this task than a single MLP network. In addition, our method ranks the 10th in the competition.

## Related Work

Reliable diagnosis of AD ought to adapt to different datasets, such as MRI scans collected by several patient groups, to reduce differences in data distribution and bias against specific groups. The existing machine learning model has been applied to the detection of AD. According to existing studies, the cortical thickness, somatotopic and hippocampal morphological features extracted by sMRI can be used to diagnose AD (Jagust, 2013). After aligning whole brain image-feature data to associate each brain voxel in common, voxel features are extracted a vector with multi-scalar measurements. The coefficients of the series are calculated and normalized to eliminate the rotation translation effect and the features used to train the SVM-based classifier. Researchers applied the gray matter voxels as input features and trained the SVM classifier to classify AD and NC categories (Klöppel et al., 2008). In practical problems, there is often more than one factor affecting a thing, that is the dependent variable corresponds to more than one independent variable. For MRI data, we should also consider more image features. However, due to the limitations of extraction methods, the data inevitably have some biases and errors that need to be corrected by humans. And traditional machine learning methods are more demanding for data processing, and different processing methods may bring large differences in results.

The existing deep learning model has been applied to the classification of AD. 2D CNN was used to extract slice features from MRI scans. Deep learning aims to reduce the use of domain expert knowledge in designing and extracting the most appropriate discriminant features (Plis et al., 2014). In the AD classification task, the researchers used a model of 3D CNN to perform feature extraction of the complete MRI, which was then used for AD/NC classification (Bron et al., 2015), and some researchers have also used unsupervised auto-encoders to pre-train convolutional layers or a more complex network to improve the accuracy of classification (Payan and Montana, 2015; Korolev et al., 2017). In some studies, part of the CNN architecture was inspired by Hosseini-Asl et al. (2018), they provide a pre-trained 3DCNN network that learns to capture generic features of AD biomarkers and adapts to datasets from different domains. There are also studies using RNN to train an AD classifier (Velazquez et al., 2019). Cheng and Liu (2017) uses extracted inter-slice features to perform the final classification. Both CNN and RNN need a large number of training data and optimized structures to achieve reliable performance. These researches used CNN-based or RNN-based to extract essential features of MRI or acquired the dense representation of MRI to build a regression model for AD score prediction or to train a different classifier. Due to CNN's or RNN's excellent performance on image classification, more researches used several data modalities on different planes and clinical scores to build multi-channel CNN and increase the model prediction ability. Although these methods perform well in image or text data, they may not be suitable for some discrete feature data, such as PRCV 2021 AD classification technology challenge dataset.

This paper uses AD classification methods based on deep learning for the PRCV 2021 AD classification technology challenge dataset, namely, SVM, RNN, CNN, region convolutional neural network (RCNN), and MLP. In order to solve the problem of the characteristics of the dataset itself and the small number of data samples, we used different MLPs to analyze the data from different atlases after screening. The advantage of this method is that it simplifies the structure of the network and prevents overfitting. At the same time, after atlas screening, some similar atlases are removed, which can reduce the negative impact of redundant data on the results. It is similar to the top-ranked methods, such as the use of multiple networks and atlas screening. In contrast to all these solutions, our method is carried out on the dataset. According to our model design and training method, the optimal model is obtained. Using the official scoring index of the competition, our model is better than other algorithms. However, due to the small number of samples in the data set, the results were somewhat unstable, and there was a gap between some optimization techniques that our team failed to surpass.

## Materials

### Datasets

The dataset was provided by the PRCV 2021 AD classification technical challenge and contains 2,600 samples. Table 1 shows the distribution and composition of the data. The age range of the samples was 32–91, with 1,982 samples concentrated between the age range of 60 and 80. The dataset contains the sample's brain gray matter volume and mean cortical thickness, which were extracted by the Computational Anatomy Tool12 (CAT12) based on multiple atlases.

**Table 1:** The distribution and composition of the data.

**Class**	**Distribution**	**Subject total**
Label	AD	671
	MCI	1,148
	NC	781
Age	Above 80	385
	60 – 80	1,983
	Under 60	232

The CAT12 software first aligns the MRI images and segments out the brain. Then, according to the different atlases, CAT12 segments the MRI and calculates the volume and cortical posteriority of the different regions. Finally, features of multiple atlases were combined to form a sequence of 28,169 one-dimensional features. These 28,169 eigenvalues are used as the feature data of this sample. Table 2 shows the information on the templates. There are 13 types and 30 versions of templates used. The name in the table indicates the name of the template, while the version indicates the version used. Each template has a different region of interest (ROI), and based on ROI, the number and value of features extracted are different.

**Table 2:** The data summary of the atlases.

**Name**	**Version**	**ROI number**
AAL (Rolls et al., 2020)	AAL(1/2/3v1)	90/116/170
AICHA (Joliot et al., 2015)	AICHA_reordered	384
Brainnetome (Fan et al., 2016)	rBN_Atlas_246_1mm	246
Brodmann (Zilles and Amunts, 2010)	Brodmann	41
Gordon (Gordon et al., 2016)	Gordon	333
Hammersmith (Hammers et al., 2003)	Hammers-mith (83/95)	83/95
Harvard-Oxford (Desikan et al., 2006)	HarvardOxford	113
Jülich (Eickhoff et al., 2005)	Juelich-thr25	103
Melbourne	Tian_Subcortex (S1/S2/S3/S4_7T)	62/54/34/16
MIST (Urchs et al., 2019)	MIST (7/12/20/36/64/122/197/325/444)	7/12/20/36/64/122/197/325/444
Scheafer (Schaefer et al., 2018)	Schaefer 2018(100/200/400/600/800/1000)	100/200/400/600/800/1000
SUIT	Cerebellum-MNIflirt	28
Yeo (Thomas Yeo et al., 2011)	Yeo2011 (7/17)	7/17

*“Version” represents the name of each version, where the different versions are indicated in “()” and “ROI Number” represents the number of ROIs for the different versions*.

## Data Preprocessing

This section introduces several novel contributions in data preprocessing. First, the atlases were filtered to reduce the dimensions when the dataset contains a small number of samples with high-dimensional morphological features. Second, the invalid value caused by the atlas mapping error was replaced by the average value or 0 when extracting the morphological feature from the brain atlas. Third, standardization was applied to adjust the data magnitude that is different between multiple atlases.

**Figure 1:**
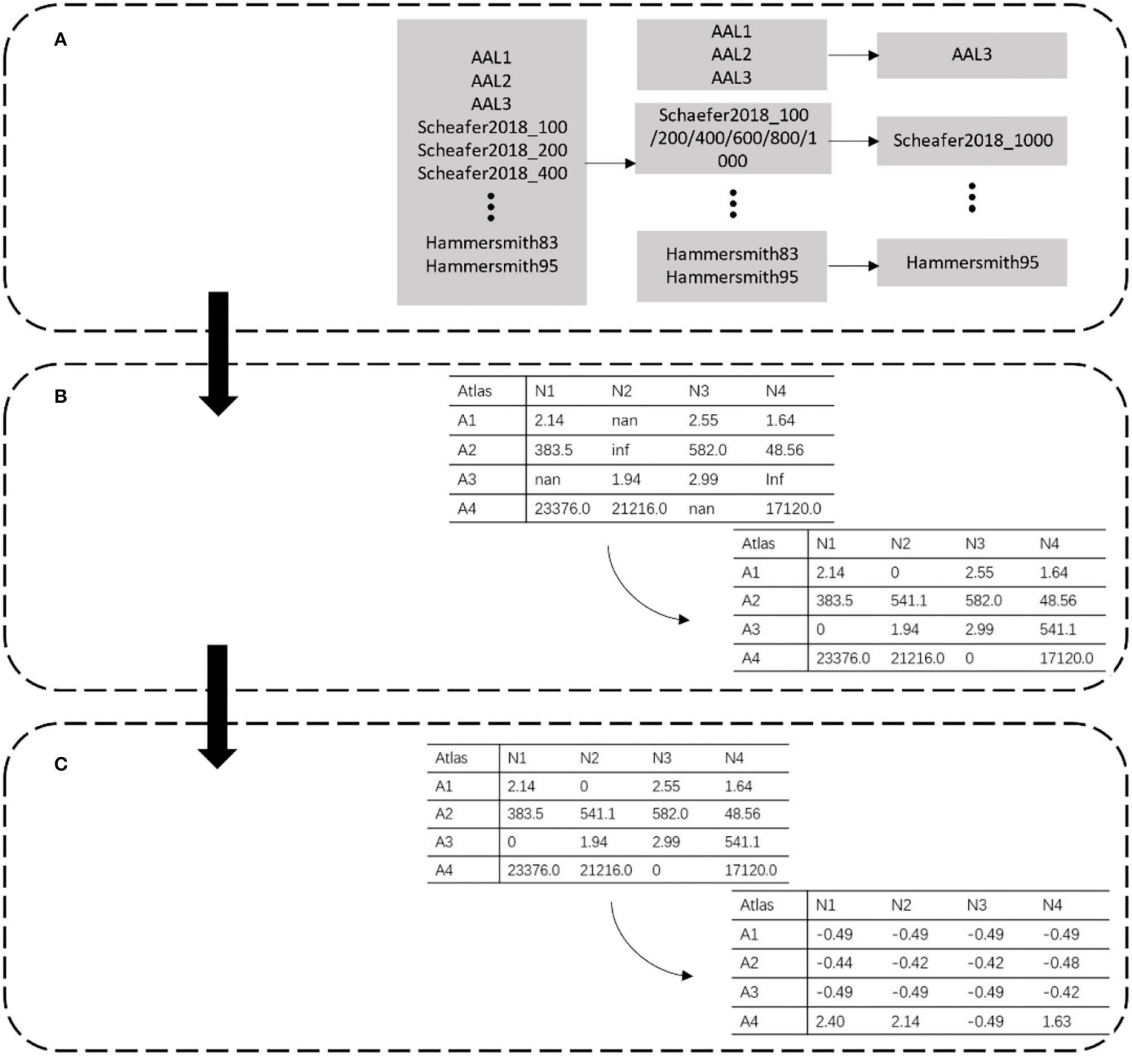
Data preprocessing. Each row represents different partitions. Atlas is represented by A, and N in each column represents the extracted data number. **(A)** Atlas filtering. **(B)** Invalid value replacement. **(C)** Data standardization.

Based on the characteristics of the dataset, the data preprocessing methods, including atlas filtering, invalid value replacement, and data normalization, were established (Figure 1).

### Atlas Filtering

The data in the PRCV 2021 AD classification technology challenge dataset combined 28,169 features extracted by 30 atlases. Among these atlases, some were similar to each other. For example, AAL1 to AAL2 to AAL3 was a process of gradual evolution and subdivision, which also had a similar relationship between Schaefer2018_1000 and Schaefer2018_100. Since a small sample with high-dimensional features caused over-fitting of the model, to reduce the feature redundancy of the template, we filtered out templates with similar functions and division basis and selected a template with the most detailed division among them (Figure 1A). For example, in the case of AAL templates, we kept the most detailed division of the AAL3v1 version as representative of this class of template. Meanwhile, we kept Schaefer2018_1000 as representative for the Schaefer2018 template.

### Invalid Value Replacement

The morphological features are extracted from the MRI image by selecting a specific brain template with the CAT12 tool. During the extraction process, part of the data was lost due to the registration error of the template, which resulted in empty and infinite values. These invalid values directly led to the disappearance of the gradient in the model during the training process. As shown in Figure 1B, we dealt with these invalid values by replacing them. Empty and infinite values were replaced with 0 and the average value, respectively.

### Data Standardization

The feature extracted from the different atlas had a magnitude difference. As shown in Figure 1C, the maximum data was >10,000, while the minimum data was <10. We standardized the data to adjust the values to the same magnitude. The mean and standard deviation of the whole dataset was calculated, and each data was divided into standard deviation from the mean. The calculation of standardized data is as follows:


(1) $$\begin{array}{l} {\hat{x}}_{ij}=\frac{x_{\overset{∙}{i}j}-mean}{std} \end{array}$$

where i is the number of data and j is the number of the eigenvalues of the data i. Mean represents the average of the dataset, and std represents the standard deviation of the dataset. Equation (2) and Equation (3) show the calculation of mean and std, respectively.


(2) $$\begin{array}{lll} m\text{e}an & = & \frac{1}{\overset{∙}{i}j}\overset{N}{\underset{i=1}{\sum }}\overset{M}{\underset{j=1}{\sum }}x_{ij} \end{array}$$

(3) $$std=\sqrt{\frac{\sum_{i=1}^{N}\sum_{j=1}^{M}x_{ij}}{\text{i}j}}$$

where the N and M are respectively, the size of the dataset and the length of each data.

After the data preprocessing, the length of data was reduced from 28,169 to 8,377. Invalid values in the data were removed by replacement. Finally, the data was standardized to reduce the gap in value.

## Methods

In this section, we present the MAMLP model using the challenge of dataset for AD prediction, specifically the one-dimensional long vector data extracted from multiple atlases. Then, we discussed the MAMLP architecture, which interlinks multiple MLP blocks with state connections, for modeling the differential information in the AD.

**Figure 2:**
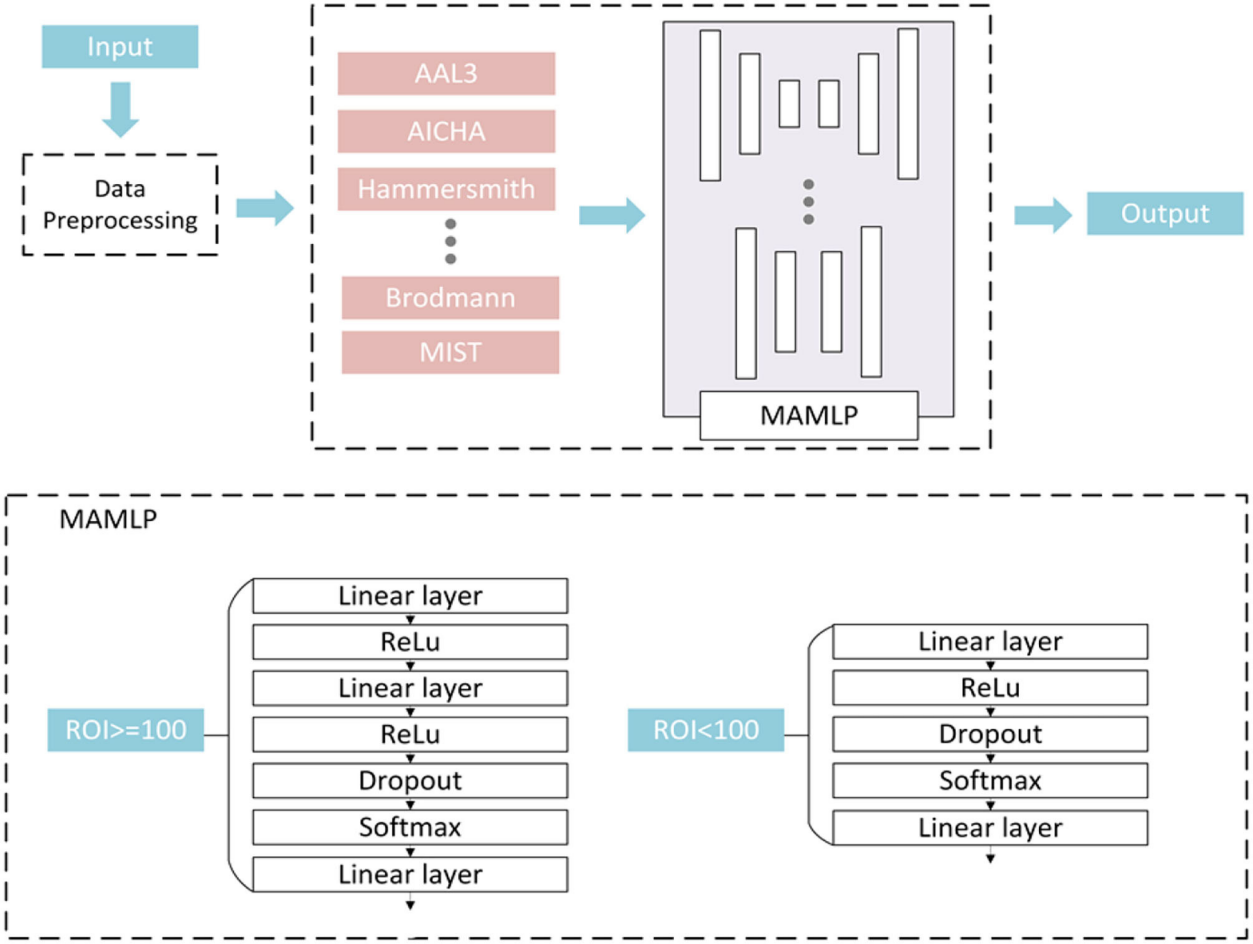
Multi-atlas multi-layer perceptron (MAMLP) Classification structure implements a two-steps scheme: three linear layers with regions of interest (ROIs) >100 and two linear layers with ROIs <100.

Further, this paper selects the data extracted from different atlases, constructs different small MLP networks according to different atlas for processing, and finally obtains the final prediction outputs combined with the results. Considering that constructing a huge MLP network often leads to overfitting due to insufficient samples of the dataset, this method not only avoided the overfitting caused by too small a sample size but also simplified the network to a certain extent and improves the efficiency of the algorithm. The structure of MAMLP is shown in Figure 2. We first separated the pre-processed data according to different atlases. The data from different atlases were input to different MLP network modules for analysis. Finally, the classification outputs of all MLP networks were combined to obtain the final result.

### Mixed Layers MLP Modular

After the separation operation, the data of different atlases were input to different MLP networks for analysis. However, we observed that the number of ROIs between the various atlases was not consistent, and the number of different feature values was extracted based on different atlases. Therefore, a fixed MLP structure Was apparently more difficult to applied to all atlases.

To solve this problem, we designed a mixed-layer MLP network to facilitate the classification, and employed a two-layer or three-layer linear layer network to process the data according to the number of each atlas. As shown in Figure 2, a fully connected network containing three linear layers was used to process the data when the number of ROIs of the atlas was >100. Unlike the three linear layers network, if the number of atlases is <100, the number of linear layers is reduced to two. In the end, different network output classification results were based on original dataset from different atlases and were combined in the subsequent operation. The final classification result of the network can be expressed by the following equation:


(4) $$O=\text{max}\left(\sum_{j=1}^{N}SoftMax(M_{j})\right)$$

where the M represents the output of the MAMLP subnetwork, j represents the jth subnetwork, and N represents the number of subnetworks.

### Ativation Function and Loss Function

In the MLP network, superscript *l* is set to represent the data related to layer *l*, which consists of L layers. The input layer is marked as 0, the output layer is marked as l, and the subscript represents the matrix or a vector index. The deactivation value of layer L is equal to the activation value of the previous layer multiplied by the network weight matrix and adds the network deviation.

Equation (5) shows the calculation method for inactive value, where *z*^*l*^ represents the inactive value of Layer *l*, *W*^*l*^ represents the layer *l* network weight matrix, and *b*^*l*^ represents the layer *l* network bias. In addition, *a*^*l*^ represents the value of the *l* layer after the activation function, and the method of calculation is shown in Equation (6), where *h*(*z*) denotes the activation function.


(5) $$\begin{array}{l} z^{l}=a^{l-1}W^{l}+b^{l} \end{array}$$

(6) $$\begin{array}{l} a^{l}=h\left(z^{l}\right)=\max\left(0,z^{l}+N\left(0,1\right)\right) \end{array}$$

Equation (5) facilitates the convergence of an end-to-end model training.

According to Equation (5) and Equation (6), *z*^*l*^ and *a*^*l*^ are calculated in order, and the output layer *z*^*L*^ is obtained. Loss function *C*(*a*^*L*^, *y*) is then calculated according to Equation (6), where *y* represents the label, and *n*^*L*^ represents the number of neurons in the output layer.


(7) $$\begin{array}{l} C\left(a^{L},y\right)=-\overset{n^{L}}{\underset{i=1}{\sum }}y_{i}\;log\;a_{i}^{L} \end{array}$$

The output ŷ of the final network is the subscript with the highest probability in *a*^*L*^. Equation (8) is the calculation method of ŷ.


(8) $$\begin{array}{l} ŷ=argmax_{i\in \left\{1,\cdots n^{L}\right\}}a^{L} \end{array}$$

Therefore, standard MLPs are not equipped to deal with unreliable input data. We show in this section that the gain of MAMLP over those models increases in two important step with unreliable inputs: multi-step prediction and dealing with original data.

### Implementation

Our approach has two key components: the first is the filtering of atlases in data preprocessing, and the second is the analysis of the network structure using different fully connected networks for different atlases. In atlas filtering, we keep the most detailed atlases among similar atlases for division. The original dataset was processed using 30 atlases for MRI and 28,169 feature values were extracted. After processing, 13 atlases containing 8,377 feature values were finally retained. In the network structure, the data were processed using a hybrid network structure.

First, we separated the data from different atlases into 13 groups and fed them into different fully connected networks for analysis. Based on the number of ROIs of the atlases, data with a number >100 features are fed into a fully connected network with three linear layers for processing. Data with a number <100 features are fed into a fully connected network with two linear layers for processing. The structure of the fully connected network with three linear layers. The first linear layer was followed by a linear rectification function (ReLU) layer as the activation function. The second linear layer is followed by a dropout layer to prevent overfitting, while the last linear layer is followed by only a Softmax layer to obtain the final classification results. The fully connected network containing two linear layers removes the first linear layer and the ReLU layer compared to the network containing three linear layers. Finally, the results of each network are combined to obtain the final classification results.

### Model Evaluation

In addition to using accuracy as the evaluation standard, we also introduce the F1 function as the evaluation index when evaluating the model. In statistics, the F1 function is used to simultaneously calculate the accuracy of unbalanced data classification problem under the consideration of the accuracy and recall of the model. The calculation formula is as follows:


(9) $$\begin{array}{l} F1=2\frac{Recall\times Precision}{Recall+Precision} \end{array}$$

In the multi-classification problem, the F1 score of each category is usually calculated first and averaged to obtain the macro F1 score. The macro F1 score is then used to evaluate the performance of the model in our experiment. The calculation formula is as follows:


(10) $$\begin{array}{l} \text{macro F}1\text{score}=\frac{{F1score}_{1}+{F1score}_{2}+{F1score}_{3}}{3} \end{array}$$

The Area Under Curve (AUC), as the evaluation index of binary classification standards, measures the ratio of true positive (recall) and false-positive classification. In multi-classification experiments, the macro F1 score is added as the evaluation index. Toward binary classification, AUC is also added to comprehensively evaluate the performance of models. The calculation formula is as follows:


(11) $$\begin{array}{l} \text{AUC}=\frac{\sum pred_{pos}>pred_{neg}}{\text{positiveNum }*\text{ negativeNum}} \end{array}$$

The denominator is the total number of combinations of positive and negative samples, while the numerator is the number of combinations where positive samples are greater than negative samples.

## Experiments and Discussion

To comprehensively evaluate the performance of the model, we set up several groups of experiments to compare and study the effects of the data dimension, network structure, and the number of atlases on the experimental results. Meanwhile, we further discuss the results of the competition and the advantages and disadvantages of our approach compared to other teams.

### Parameter Setting of Experiment

The experimental environment of this paper was the PyTorch framework and NVIDIA–TITAN-XP GPU. During the training process, we adopted the following strategies: Cross-Entropy as the loss function; Stochastic Gradient Descent (SGD) as the optimizer; the learning rate is set to 0.001; the dropout layer in the network is set to 0.5. We divided the number of the training-set and test-set into 2,300:300, and 100 cases of each label were selected in the test-set. In the AD/NC/MCI experiment, four indicators were used for evaluation, including Accuracy, Precision, Recall, and F1score. AUC was used as an evaluation indicator in the binary classification experiment. The higher all the indicators, the better the effect of classification.

### Comparative Experiments of Data Pre-processing

In the data pre-processing section, the following pre-processing operations are performed on the data: (1) Atlas Filtering for feature dimension reduction; (2) replacement of invalid values in the data; and (3) standardization of the data values. To demonstrate the effectiveness of these treatments, we conducted comparative experiments on data pre-processing.

Table 3 shows the impact of data pre-processing on the experiment. Compared with the unfiltered data and unstandardized data, the accuracy of the pre-processed data is improved greatly. These experiments were performed by default after the second pre-processing operation (invalid value replacement) because the model would have experienced gradient disappearance without this preprocessing. The results of the experiments show that (1) “Filtering Atlas” had an impact on the accuracy of the model, improving it by about five percent; and (2) “Numerical standardization” is significant. Without standardization, differences in extraction criteria between templates will make it difficult for the model to learn valuable information.

**Table 3:** Effect of data preprocessing on the experiment.

**Preprocessing**	**Accuracy**	**Precision**	**Recall**	**F1 score**
Without preprocessing	0.25	0.15	0.11	0.13
Filtering atlas	0.51	0.47	0.43	0.45
Data standardization	0.64	0.63	0.64	0.64
Data standardization and filtering atlas	**0.67**	**0.68**	**0.67**	**0.68**

*All experiments assume that the invalid values have been removed. Otherwise, the experiments cannot be performed. Bold values mean the best value in the comparative experiment*.

Considering that a huge number of atlases are used in the data extraction process and that some atlases have high similarities, we filtered the models in the data preprocessing stage and selected one in the similar atlases. To further explore the accuracy of the model with the different number of atlases, we tried to keep more atlases or further removed them.

**Figure 3:**
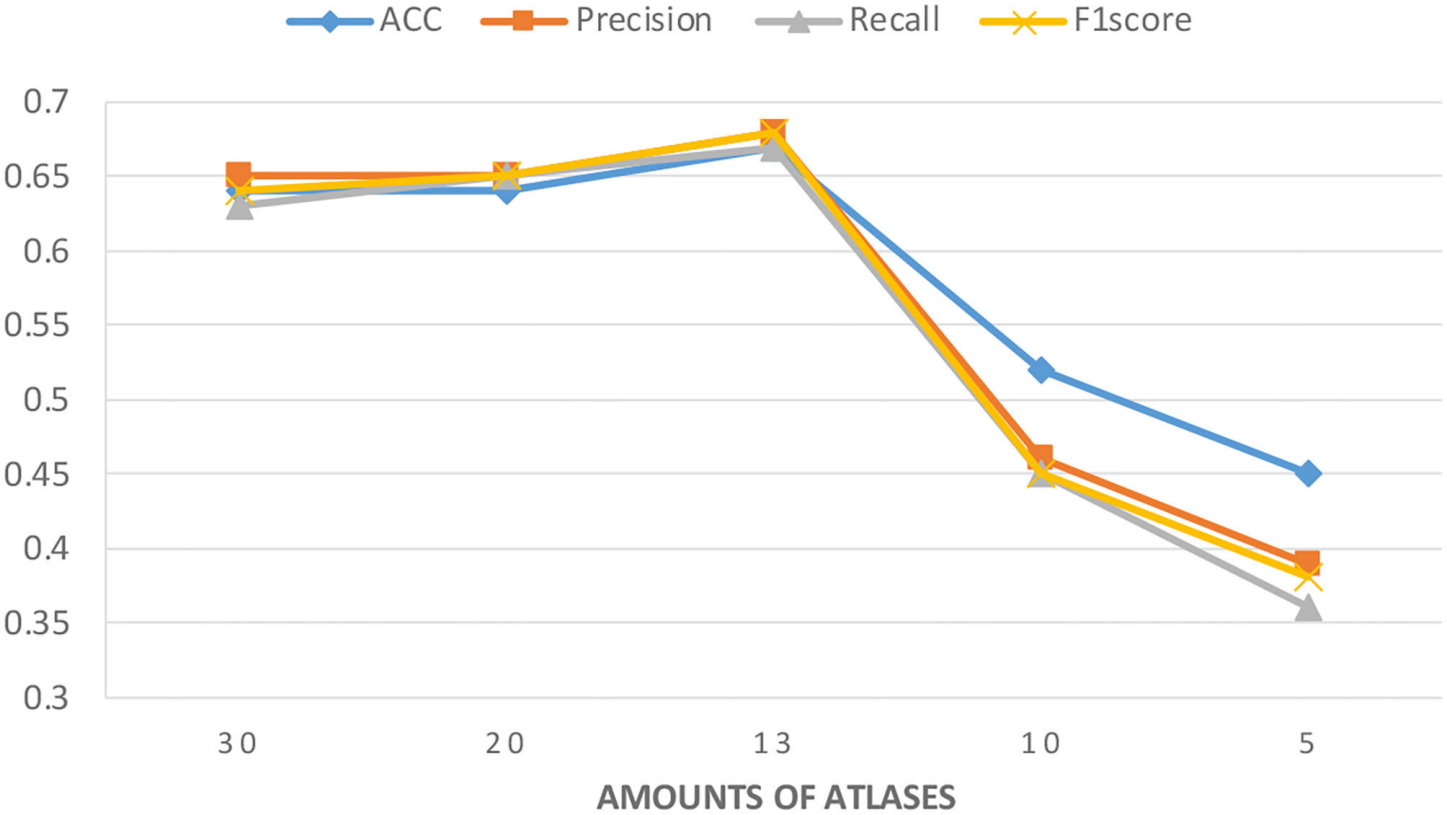
Classification accuracy of the model under different amounts of atlases. The number of atlases after data preprocessing is 13.

Figure 3 shows the experimental results with different numbers of atlases. The number of atlases after data preprocessing is 13. These results suggest that the classification accuracy was improved by removing similar atlases, but the classification accuracy showed a decreasing trend when atlases are further removed. These findings are understandable because using too many similar atlases causes the number of features per sample to exceed the sample size of the PRCV 2021 AD classification technology challenge dataset. A situation that over-fits the model while using too few atlases does not provide sufficient feature data. Therefore, choosing the appropriate number of atlases can further improve the classification accuracy of the model.

### Different Methods Based on Different Data Dimensions

By splicing the data, the original one-dimensional data can be spliced into two- or three-dimensional data. Then, the convolution under the corresponding dimension can be used for data processing and analysis. We follow that these extracted data do not have image characteristics, such as color and form. Therefore, the method of using convolutional analysis after up dimensioning is considered to have poor performance for PRCV 2021 AD classification technology challenge dataset. We processed the data as two-dimensional and three-dimensional fake-image data and used classical CNN to process them.

In the experiment of different dimensions, the data are spliced as 168×168 two-dimensional data and 31×31×30 three-dimensional data. The data is then processed by invalid value replacement and standardization before training. On the two-dimensional data, visual geometry group (VGG) (Simonyan and Zisserman, 2014) and ResNet50 (He et al., 2016) are used to analyze the data, while the full-size diagnosis network (FDN) is used on the three-dimensional data (Li et al., 2019). For the comparison experiments, we use the same learning rate and batch size. The network structure is also the same as in the original paper, except that the FDN model uses a non-iterative version. The setup of these methods follows the original design of their papers. Meanwhile, we use an MLP network with 4 linear layers to compare with our method and evaluate the effectiveness of the method in four metrics, which are Accuracy, Precision, Recall, and F1score.

As shown in Table 4, our method obtained the best results in all four metrics. In addition, the method of raising the dimensionality does not effectively improve the classification accuracy. These results suggest that the method of using CNN for feature extraction on two-dimensional or three-dimensional data is not as effective as the method of using MLP on one-dimensional data. These findings are understandable because although the data has been improved on the dimension, it still does not have image features, such as color-feature or shape-feature. In addition, the CNN still cannot extract those disease-related features well. Compared with a single MLP network, since the data extracted from different atlas are not correlated, our method separates them and uses different networks for analysis, which can better prevent model overfitting and prevent mutual interference between different atlas data.

**Table 4:** Data summary of different methods based on different data dimensions.

**Data dimension**	**Models**	**Accuracy**	**Precision**	**Recall**	**F1 score**
One-dimensional	MLP	0.66	0.66	0.65	0.66
	MAMLP (ours)	**0.67**	**0.68**	**0.67**	**0.68**
Two-dimensional	VGG	0.56	0.58	0.55	0.56
	ResNet50	0.64	0.66	0.61	0.62
Three-dimensional	FDN	0.55	0.53	0.54	0.55

*The bold part represents the best result*.

### Different Methods Based on One-Dimensional Data

One-dimensional feature data in the PRCV 2021 AD classification technology challenge dataset comes from gray matter volume and mean cortical thickness components extracted from different atlases. Unlike MRI, the data in the dataset loses original image characteristics, such as color or shape. The methods which are used to process MRI on two-dimensional or three-dimensional had poor performance for this dataset. However, some methods for natural language processing are often used to process one-dimensional feature data. Hence, we compared these methods with ours.

The procedure we followed can be briefly described as data pre-processing using different methods to analyze the data and four indicators to evaluate the model. We use three methods to compare with our method, including CNN-1d, RNN, and RCNN (Kim, 2014; Liu et al., 2016; Zhou et al., 2016).

As shown in Table 5, our method obtained the best results in all four metrics. The research we have done suggests that these natural language processing-related methods are not very good at extracting the relationship between features and disease stages compared to our methods. The CNN has advantages in performing two-dimensional image feature extraction, but does not work well for processing one-dimensional long vector data. Recurrent neural networks are mainly concerned with the temporal relationship between features and perform poorly in identifying the relationship between features and classification results. For PRCV 2021 AD classification technology challenge dataset, it has lost its original imaging features after atlas extraction, and the correlation between each feature is not obvious. As a result, CNN and RNN-related methods do not apply to this dataset compared to MLP.

**Table 5:** Data summary of in Alzheimer's disease (AD)/mild cognitive impairment (MCI)/normal control (NC) classification.

**Methods**	**Accuracy**	**Precision**	**Recall**	**F1 score**
CNN-1d (Kim, 2014)	0.55	0.52	0.52	0.52
RNN (Liu et al., 2015)	0.65	0.64	0.63	0.64
RCNN (Zhou et al., 2016)	0.62	0.62	0.62	0.63
MAMLP (ours)	**0.67**	**0.68**	**0.67**	**0.68**

*The bold part represents the best result*.

To further measure the performance of the model, we take AUC as the evaluation standard and experiment on binary classification problems. Among them, the number of samples in each category in the classification problems of NC/MCI, NC/AD, and MCI/AD are 781/1,148, 781/671, and 1,148/671, respectively. We divide the train set and test set according to a ratio of 4:1. For the rest of the setup, it was kept consistent with the triple classification experiment.

Table 6 shows that the performance of the four methods in the three binary classification tasks. In the classification of NC/MCI and NC/AD, our model obtained the highest score. RNN model performs better in the classification of MCI/AD. In the experiments with dichotomous classification, the performance of the individual models was largely consistent with that of trichotomous classification, but in MCI/AD, the RNN performed much better. This phenomenon illustrates that our method is more sensitive to the differences between NC and AD/MCI and is more accurate in determining whether the disease is present.

**Table 6:** Data summary of different models in binary classification.

**Methods**	**Accuracy**	**F1 score**	**AUC**
**(a) Data summary of NC/MCI classification**			
CNN-1d (Kim, 2014)	0.65	0.75	0.60
RNN (Liu et al., 2015)	0.73	0.79	0.71
RCNN (Zhou et al., 2016)	0.73	0.80	0.69
MAMLP (ours)	**0.75**	**0.81**	**0.74**
**(b) Data summary of NC/AD classification**			
CNN-1d (Kim, 2014)	0.84	0.83	0.84
RNN (Liu et al., 2015)	0.86	0.84	0.85
RCNN (Zhou et al., 2016)	0.85	0.83	0.84
MAMLP (ours)	**0.89**	**0.89**	**0.90**
**(c) Data summary of MCI/AD classification**			
CNN-1d (Kim, 2014)	0.68	0.69	0.64
RNN (Liu et al., 2015)	**0.78**	**0.71**	**0.77**
RCNN (Zhou et al., 2016)	0.77	0.68	0.75
MAMLP (ours)	0.77	0.66	0.74

*The bold part represents the best result*.

Meanwhile, we compared the differences between the fixed MLP network and the hybrid MLP network, which is to verify whether this approach can improve the classification accuracy. As shown in Table 7, the mixed network structure exhibits a greater advantage in all metrics compared to the fixed one. This phenomenon is also easily explained by the fact that a small network is not suitable for large inputs when approaches use a fixed network structure and vice versa. If a fixed structure is used in all MLP sub-networks, the number of features per template should be fixed, which is difficult to achieve. Therefore, a mixed network structure is a more suitable method.

**Table 7:** Different numbers of linear layers on multi-layer perceptron (MLP) modules.

**Number of linear layers**	**Accuracy**	**Precision**	**Recall**	**F1 score**
Two	0.56	0.51	0.52	0.52
Three	0.59	0.58	0.57	0.58
Two and three mixed	**0.67**	**0.68**	**0.67**	**0.68**
Four	0.58	0.59	0.58	0.59

*The bold part represents the best result*.

**Figure 4:**
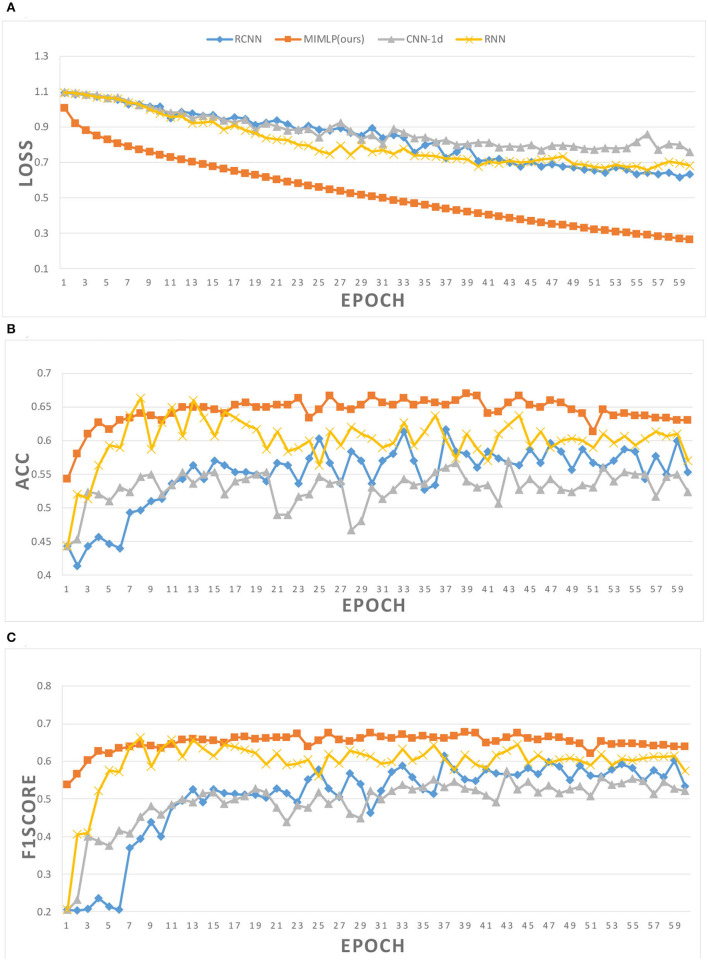
Training details of different models. **(A)** Loss in training. **(B)** Accuracy in training. **(C)** F1score in training.

In addition, we believe that there is no correlation between data from different atlases. Different from the original MLP network, referring to the Ortiz's method (Ortiz et al., 2016), the data is segmented according to different atlases and then sent into different MLP models for classification before the results are combined. In this way, we effectively reduce the complexity of the model and prevent the overfitting of the algorithm. Figure 4 shows the change process of loss, accuracy, and f1score in the training process of different models. With the continuous improvement of training times, the value of loss continues to decline while the classification results of some models gradually deteriorate. It can be inferred that due to the small sample size and excessive training, the model has the phenomenon of overfitting, which is more obvious in the complex model. Compared with other models, our model performs better in both the convergence speed of loss and the ability to prevent overfitting.

### Discussion of PRCV2021

**Figure 5:**
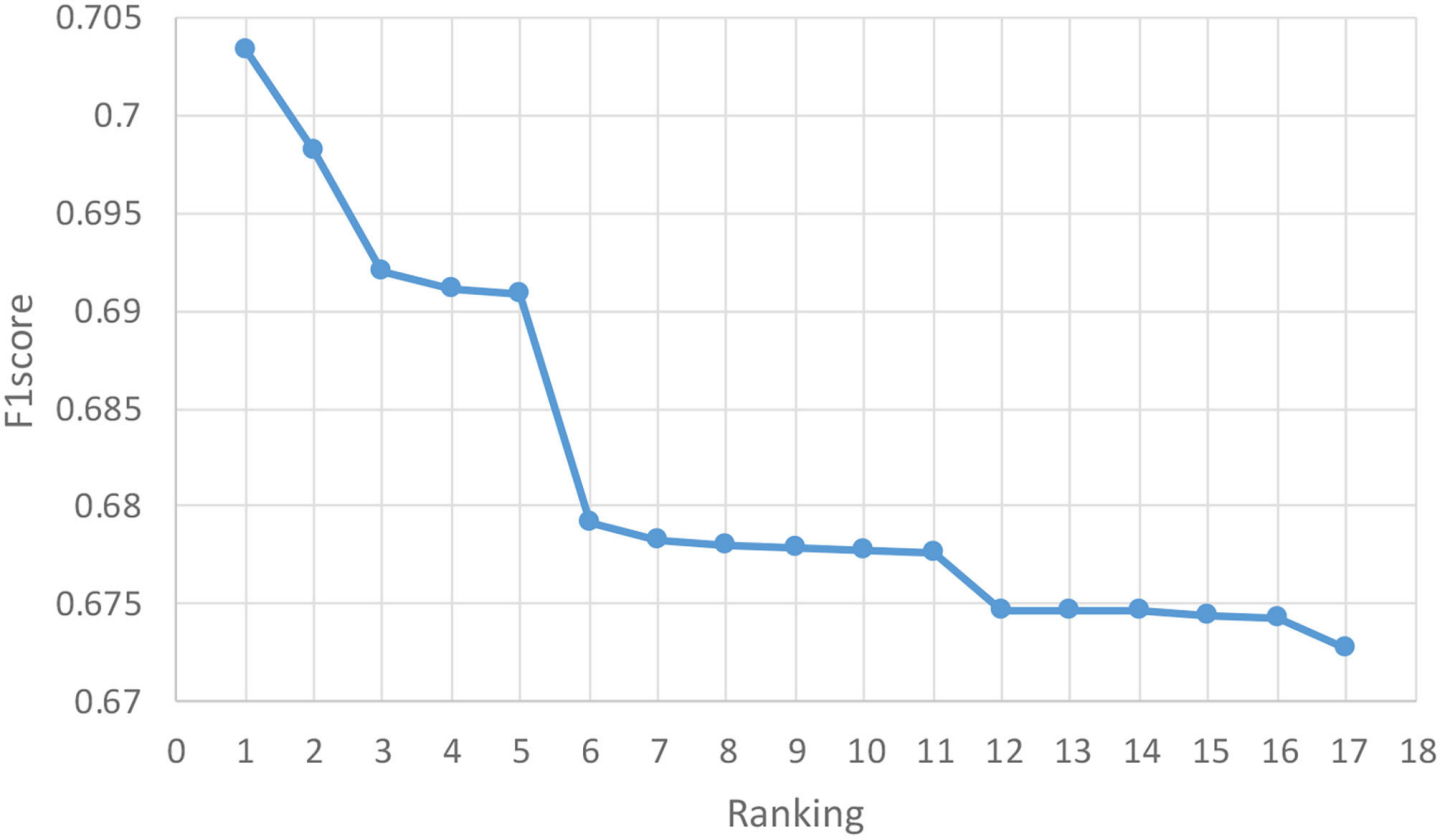
Results of the competition. The horizontal axis represents the ranking of the competition and the vertical axis represents the score of the competition. The competition uses F1score as the final evaluation metric. The figure shows the 17 winning teams among 373 teams, among which our team gained the rank of 10^th^.

PRCV 2021 AD Classification Technical Challenge provides a dataset containing gray matter volumes and mean cortical thickness extracted from multiple atlases. Based on this dataset, PRCV 2021 proposes a triple classification task for AD. Figure 5 shows the rankings and scores of all winning teams in the competition, among which our team ranks 10th. Most of the better performing teams in the competition have optimized their methods based on the MLP architecture. The adjustments on the network are as follows: combining MLP with attention mechanism, adjusting the depth of MLP network, and combining multiple networks for data processing, etc. For the processing of the dataset, some teams filtered the data based on the characteristics of the atlas or supplemented the data by interpolation.

In the competition, most of the teams used the MLP-based network and did various optimized operations. Among them, the best-performing method used a combination of MLP and attention and got the highest score of 0.7033. They added multiple attention modules to the network and connected outputs of different depths as input to the module. Compared with their method, we all used multiple different MLPs for training. The advantage of this is that it can effectively avoid the uncertainty of classification accuracy under a single model. However, their method adds an attention mechanism before obtaining the classification results so that the model can more accurately identify the characteristics related to the disease type and reduce the interference of other redundant data to solve the problem of overfitting.

There were also teams in the competition that used traditional machine learning algorithms, mainly random forests and SVM, and achieved good results. We think that traditional machine learning algorithms are also very applicable to this type of data. However, through post-competition experience sharing, we found that most of the machine learning teams focused their work on data processing and that most of the teams that won awards had a good approach to processing the dataset. Hence, in that task, the machine learning algorithms had higher requirements for data processing compared to deep learning related methods.

Similar to our method was that of the team that won fifth place. They also used different MLP networks to train data from different atlases. However, the difference is that our method removes some similar atlases before training, while their method selects the atlas based on the training results after training. After an analysis, we believe that their method is more appropriate because the correlation between the extracted results of the atlas and the disease should be judged by the model.

Compared to teams with similar scores to ours, our method still has a certain advantage. For example, the seventh-place team uses a clever way to optimize. They train a large number of networks, and select the four with the best results to combine. Due to the small number of samples and larger number of feature values in the PRCV 2021 AD classification technology challenge dataset, most of the teams' methods suffer from overfitting problems. This is also evident in the training process, where the same model and parameters end up with a significant difference in classification accuracy. They take advantage of this feature to train a model that better fits the test set. Although this method has obtained good scores in the competition, its performance may not be good if the test set is re-divided. Compared with their method, our method is more versatile.

## Conclusion

Against the dataset provided by the PRCV 2021 AD classification technology challenge, we propose a MAMLP model for Alzheimer's classification based on brain region data extracted by multi-atlas segmentation. The results of the experiment indicate that our model has better classification accuracy and generalization ability when targeting such datasets. Of course, our method is not optimal, as there are similarities in the ideas of the method compared to the teams ranked before us. For example, redundant data are removed by atlas selection and multiple networks are used for combination. The disadvantage is the lack of skill in training or the randomness caused by the small sample. An obvious limitation of this study is that the overfitting of the model due to the small sample has not been fully resolved. The next step is to use some small sample training methods to further improve the accuracy of the model. At the same time, compared with other teams' data processing methods, our method still has some gaps. In the face of high-dimensional data, dimensionality reduction is an important step, and if we can effectively remove some redundant data and duplicate data, we believe the classification effect of the model can become better.

## Data Availability Statement

Publicly available datasets were analyzed in this study. This data can be found at: https://competition.huaweicloud.com/information/1000041489/circumstance.

## Author Contributions

XH supervised and managed the study, guided the experiment, and revised the manuscript. KH and JL performed the experiments, processed the data, wrote the manuscript, and were jointly responsible for its revision. XY, EC, and LC provided the technical support in the writing of the article. GW and SZ were responsible for the revision of the manuscript. All authors have read the manuscript and agreed to the published version.

## Funding

This work was supported by Fuzhou science and technology planning Project: Development and application of intelligent management technology of Cambodian national chronic disease (No: 2020-DY-185). The funder was not involved in the study design, collection, analysis, interpretation of data, the writing of this article or the decision to submit it for publication.

## Conflict of Interest

XY was employed by Fuzhou Comvee Network and Technology Co., Ltd. The remaining authors declare that the research was conducted in the absence of any commercial or financial relationships that could be construed as a potential conflict of interest.

## Publisher's Note

All claims expressed in this article are solely those of the authors and do not necessarily represent those of their affiliated organizations, or those of the publisher, the editors and the reviewers. Any product that may be evaluated in this article, or claim that may be made by its manufacturer, is not guaranteed or endorsed by the publisher.

## Footnotes

^1^Pattern Recognition and Computer Vision 2021 Alzheimer's disease classification technology challenge: https://competition.huaweicloud.com/information/1000041489/circumstance.
